# Supplementary material for: 2.7 Å cryo-EM structure of human telomerase H/ACA ribonucleoprotein
Source: Nat Commun. 2024 Jan 25;15:746. doi: 10.1038/s41467-024-45002-x (PMC10811338; doi:10.1038/s41467-024-45002-x)
Supplement: Supplementary file 1 — Supplementary Information [file 41467_2024_45002_MOESM1_ESM.pdf]

Supplementary Information for

2.7 Å cryo-EM structure of human telomerase H/ACA ribonucleoprotein

George E. Ghanim[1], Zala Sekne[1], Sebastian Balch, Anne-Marie M. van Roon and Thi Hoang Duong Nguyen*

MRC Laboratory of Molecular Biology, Cambridge CB2 0QH, UK.

[1] These authors contributed equally: George E. Ghanim, Zala Sekne.

* To whom correspondence should be addressed. Email: knguyen@mrc-lmb.cam.ac.uk (T.H.D.N.).

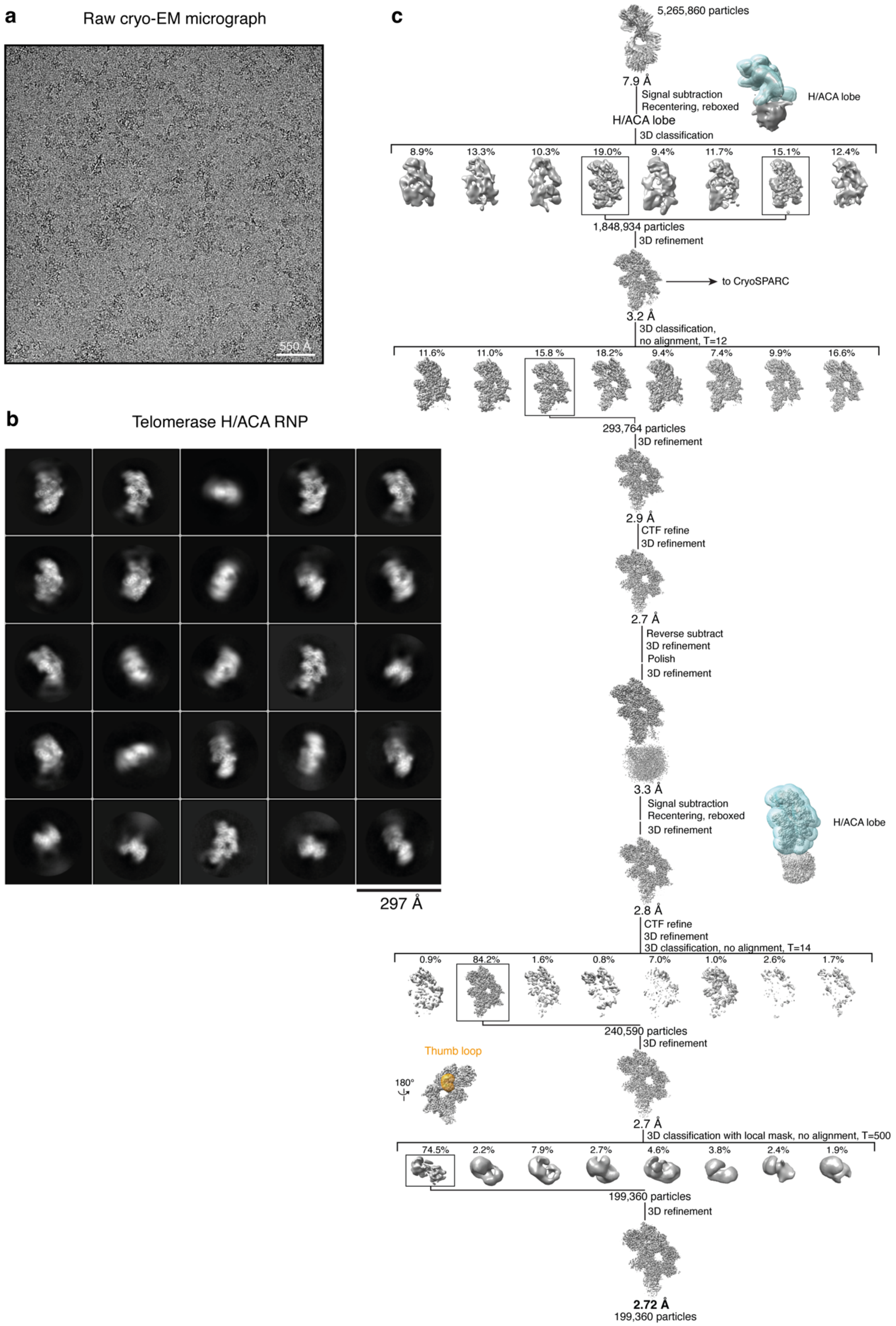
a
Raw cryo-EM micrograph
550 Å
b
Telomerase H/ACA RNP
297 Å
c
5,265,860 particles
7.9 Å
Signal subtraction
Recentering, reboxed
H/ACA lobe
H/ACA lobe
3D classification
8.9% 13.3% 10.3% 19.0% 9.4% 11.7% 15.1% 12.4%
1,848,934 particles
3D refinement
to CryoSPARC
3.2 Å
3D classification,
no alignment, T=12
11.6% 11.0% 15.8 % 18.2% 9.4% 7.4% 9.9% 16.6%
293,764 particles
3D refinement
2.9 Å
CTF refine
3D refinement
2.7 Å
Reverse subtract
3D refinement
Polish
3D refinement
3.3 Å
Signal subtraction
Recentering, reboxed
3D refinement
H/ACA lobe
2.8 Å
CTF refine
3D refinement
3D classification, no alignment, T=14
0.9% 84.2% 1.6% 0.8% 7.0% 1.0% 2.6% 1.7%
240,590 particles
3D refinement
Thumb loop
180°
2.7 Å
3D classification with local mask, no alignment, T=500
74.5% 2.2% 7.9% 2.7% 4.6% 3.8% 2.4% 1.9%
199,360 particles
3D refinement
2.72 Å
199,360 particles

Supplementary Fig. 1: Cryo-EM data processing workflow for the consensus refinement of human telomerase H/ACA RNP lobe. In the resulting map, the thumb loop of the 3' dyskerin adopts a "semi-closed" conformation. **a** Representative cryo-EM micrograph. **b** Cryo-EM 2D class averages of the H/ACA RNP after signal subtraction and recentering with a box size of 280^2 pixel. **c** Image processing workflow that yielded the 2.7 Å reconstruction of the telomerase H/ACA RNP.

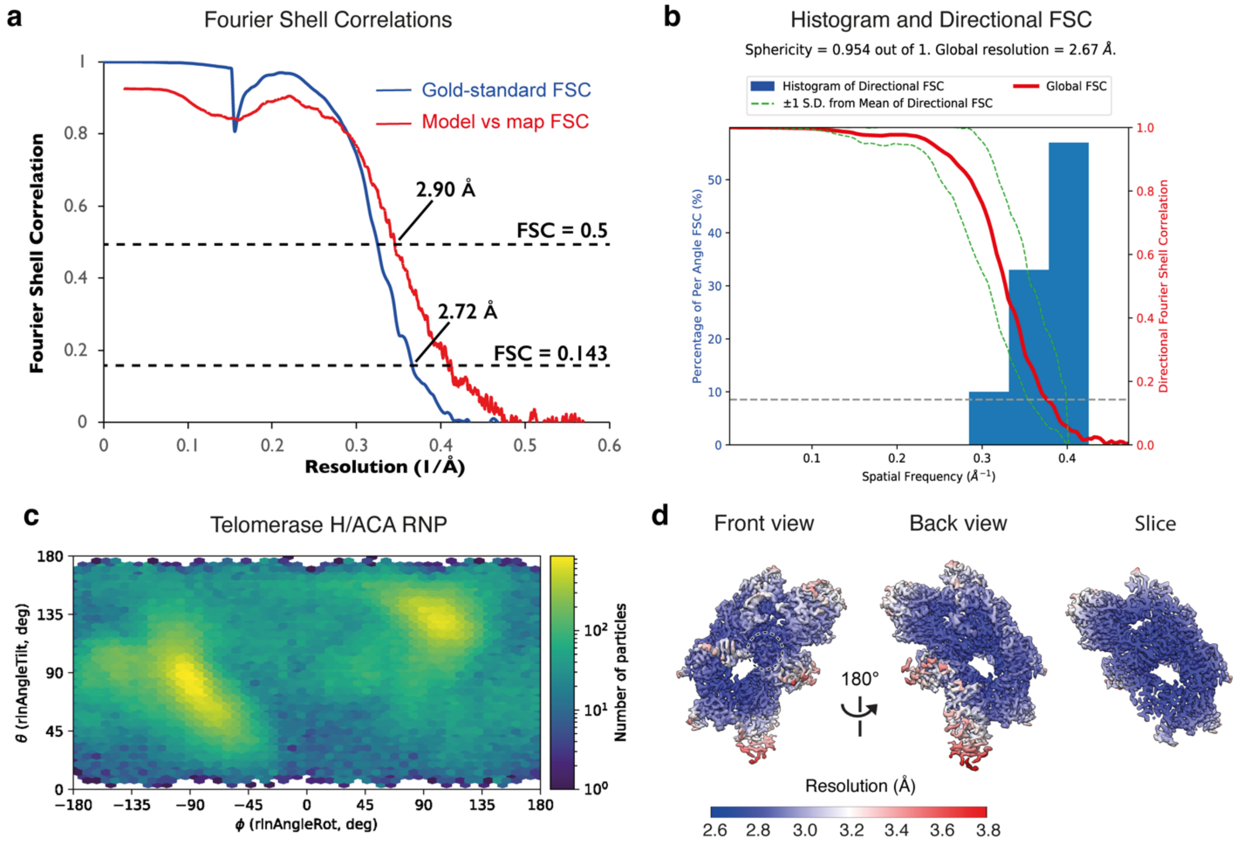

Supplementary Fig. 2: Overall and local resolution estimation for the 2.7 Å consensus map of telomerase H/ACA RNP lobe. a Gold-standard (blue) and model-vs-map (red) FSC plots for telomerase H/ACA RNP reconstruction. Resolution was estimated at FSC = 0.143 (gold-standard) and at FSC = 0.5 (model-vs-map). **b** Directional FSC plots and sphericity values for telomerase H/ACA RNP reconstruction. A 3D-FSC server (https://3dfsc.salk.edu/) was used to calculate directional FSC plots. **c** 2D histograms displaying the Euler angles covered by particles used for the reconstruction of the telomerase H/ACA RNP. A Python script (https://githubhelp.com/Guillawme/angdist) was used to calculate the 2D histograms. **d** Local resolution of the consensus map of telomerase H/ACA RNP. Local resolution estimation was obtained by RELION[1] and displayed in UCSF ChimeraX. The position of the thumb loop of the 3' dyskerin is indicated with a circle.

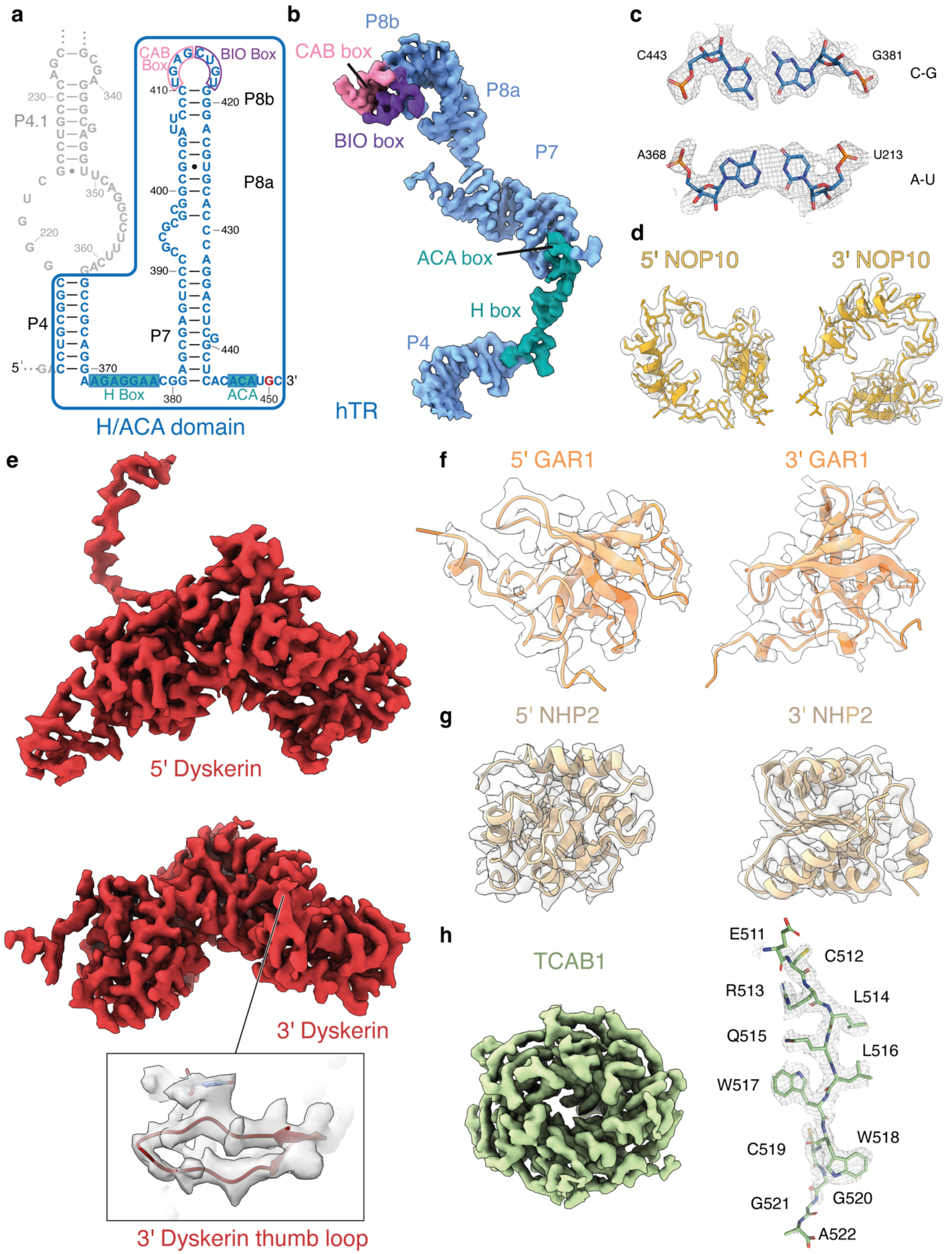

Supplementary Fig. 3: Representative cryo-EM densities of the 2.7 Å reconstruction of telomerase

H/ACA RNP. a Secondary structure of the H/ACA domain of hTR. Nucleotide mutated in Supplementary Fig. 9 is shown in red. **b** Full density of hTR. **c** Example of EM densities of a G-C and an A-U base pairing. In most of the map, densities for different bases are clearly discernible. **d–h** Full densities of NOP10 (**d**), dyskerin (**e**), blurred 3' dyskerin thumb loop (**e, inset**), GAR1 (**f**), NHP2 (**g**), and TCAB1 (**h**). **h** Representative EM densities of TCAB1 (E511-A522), respectively.

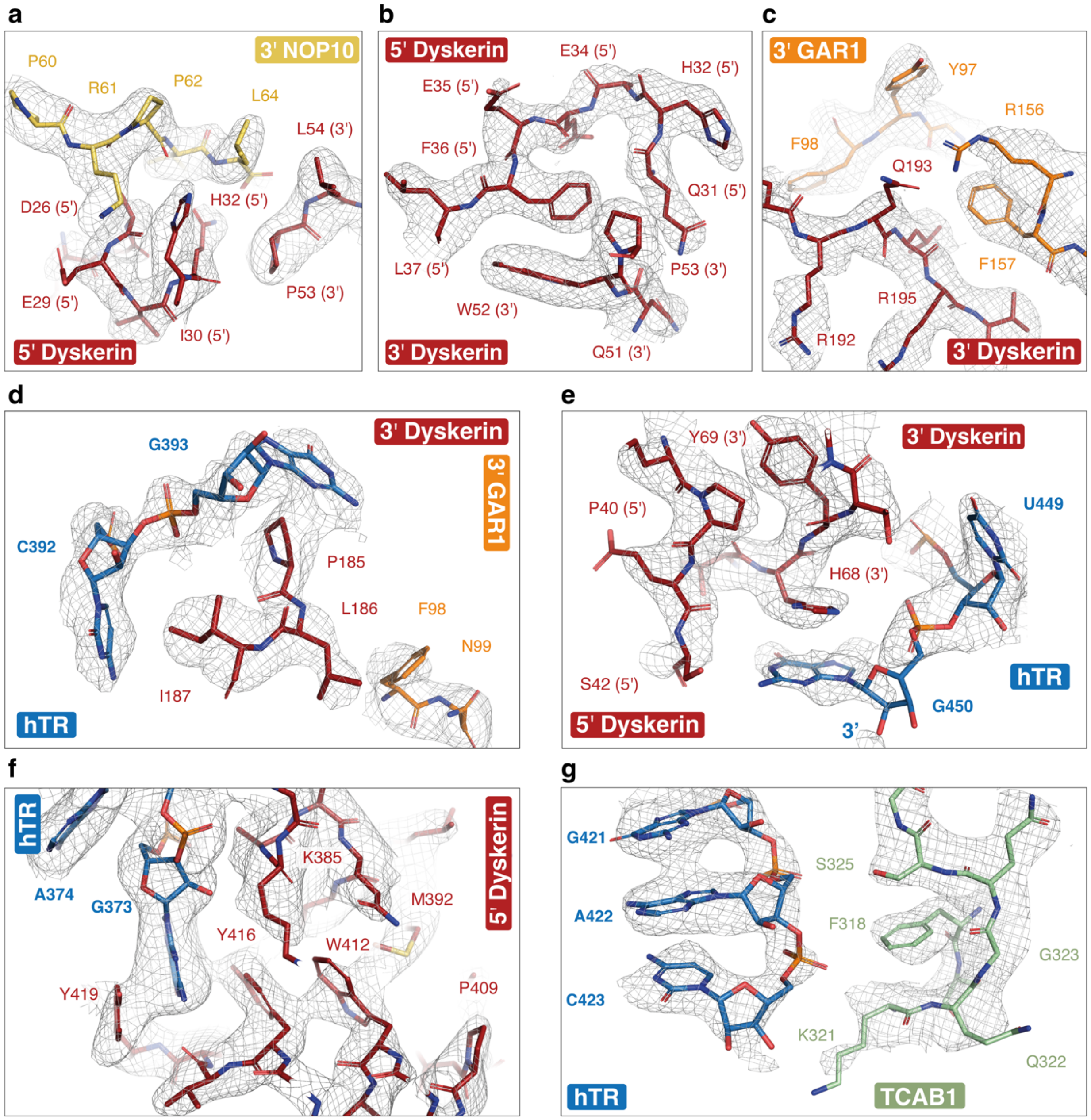

Supplementary Fig. 4: Cryo-EM densities of regions from the consensus 2.7 Å telomerase H/ACA RNP map discussed in the main text. **a** Interactions between N-terminal helix 1 of the 5' dyskerin and the C-terminus of the 3' NOP10 (also see Fig. 3c). **b** Interactions between the N-terminal regions of the 5' and 3' dyskerin (also see Fig. 2f). **c** Interactions between the 3' dyskerin thumb loop and the 3' GAR1 (also see Fig. 6c). **d** The 3' dyskerin thumb loop and its interaction with hTR and the 3' GAR1 (also see Fig. 6b). **e** Interactions between the 3' end of hTR, the 5' dyskerin and the 3' dyskerin (also see Fig. 4f). **f** Interactions between the H box of hTR and the 5' dyskerin CTE (also see Fig. 4c). **g** Interactions between the P8 stem-loop of hTR and TCAB1 (also see Fig. 5c).

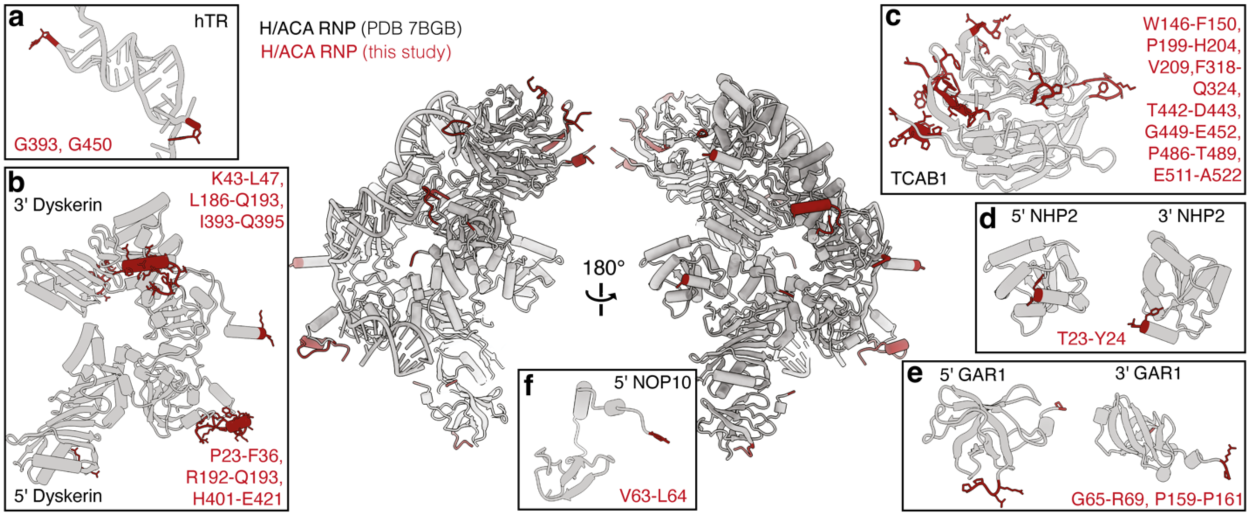

Supplementary Fig. 5: Newly resolved regions in the telomerase H/ACA RNP. The atomic model for the telomerase H/ACA RNP with newly resolved regions highlighted in maroon. The panels present **a** hTR, **b** dyskerin, **c** TCAB1, **d** NHP2, **e** GAR1 and **f** NOP10, respectively. The newly built residues are showed as sticks.

a

Dyskerin sequence

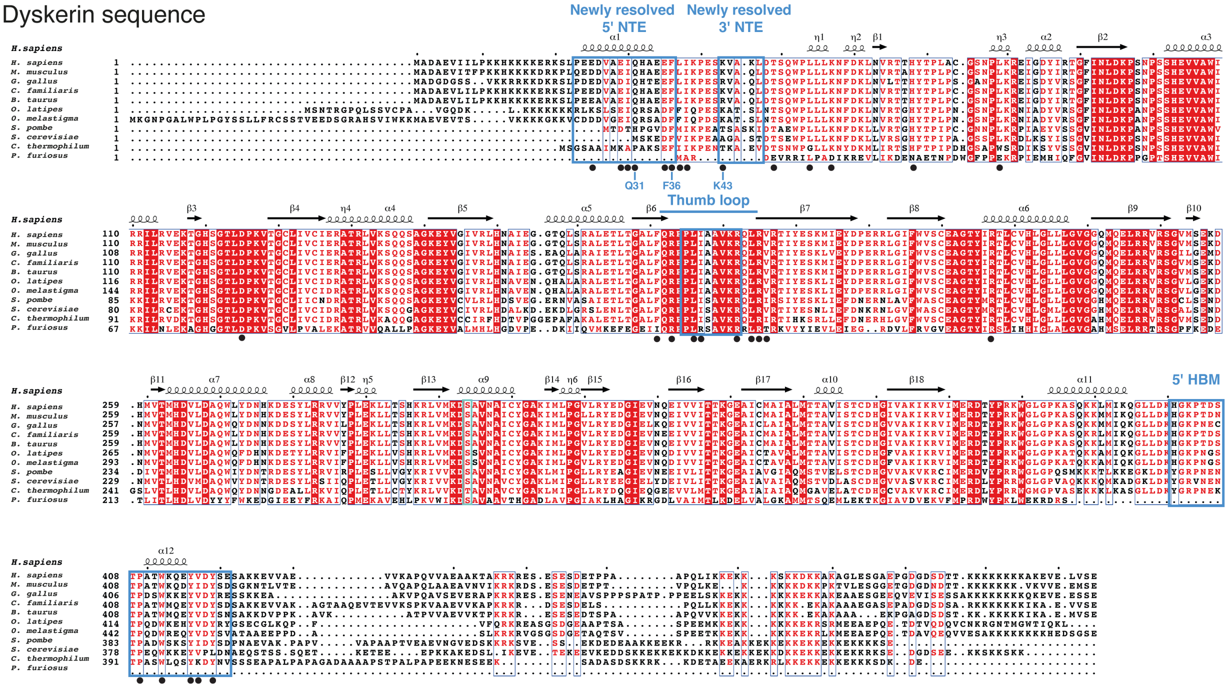

b

GAR1 sequence

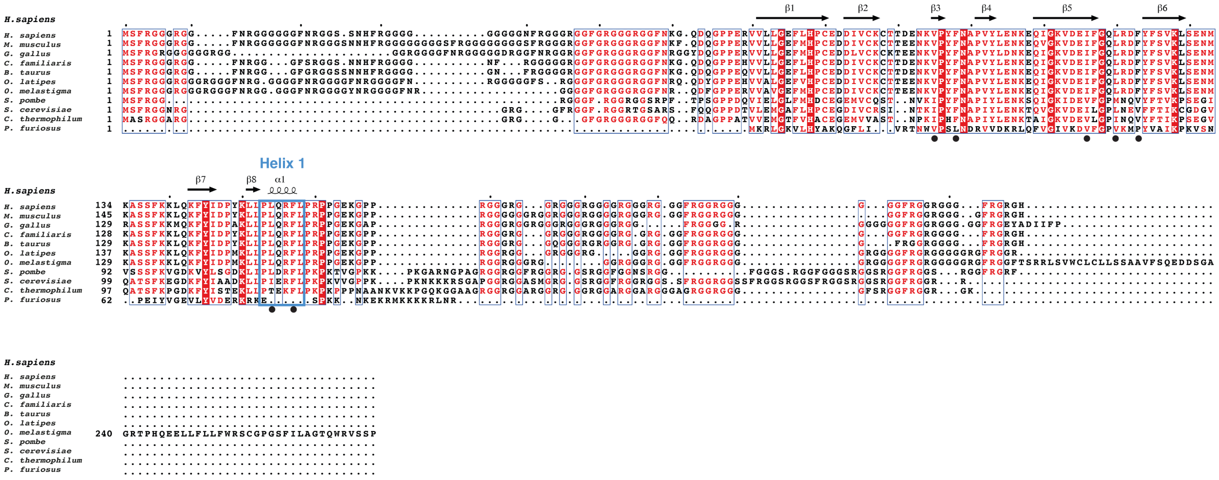

c

NHP2 sequence

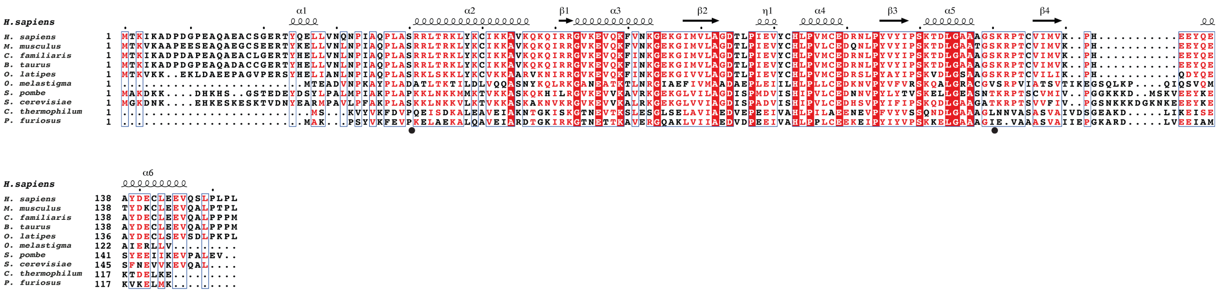

d

NOP10 sequence

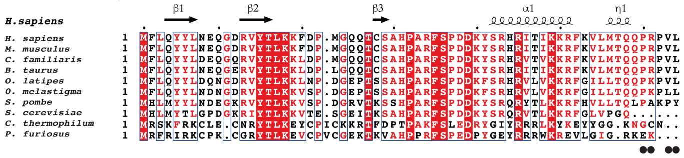

e

TCAB1 sequence

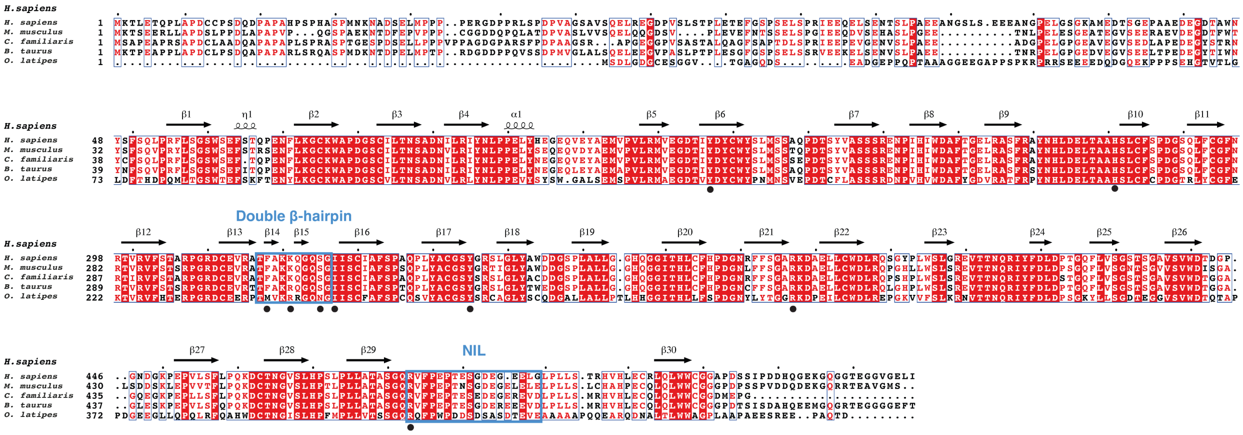

Supplementary Fig. 6: Sequence alignments of telomerase H/ACA RNP subunits. Sequence alignments of **a** dyskerin, **b** GAR1, **c** NHP2, **d** NOP10 and **e** TCAB1 with their homologues from eleven species (10 for NHP2 and NOP10, 5 for TCAB1) with representatives from vertebrates, fungi, cilia and archaea. Sequences were obtained from UniProt and aligned using Clustal Omega server[2]. Visualization was done using ESPript 3.0[3]. Residues with sequence conservation higher than 70% are colored. Red highlights indicate absolute conservation. Secondary structures of dyskerin, GAR1, NHP2, NOP10 and TCAB1 obtained from our structure are shown at the top of each sequence. Residues discussed in the main text are labelled with a black dot. Newly resolved NTE of dyskerin, parts of the dyskerin thumb loop, the HBM of the 5' dyskerin, the NIL and β-hairpin loops of TCAB1, and the CTE helix 1 of GAR1 are highlighted with blue rectangles.

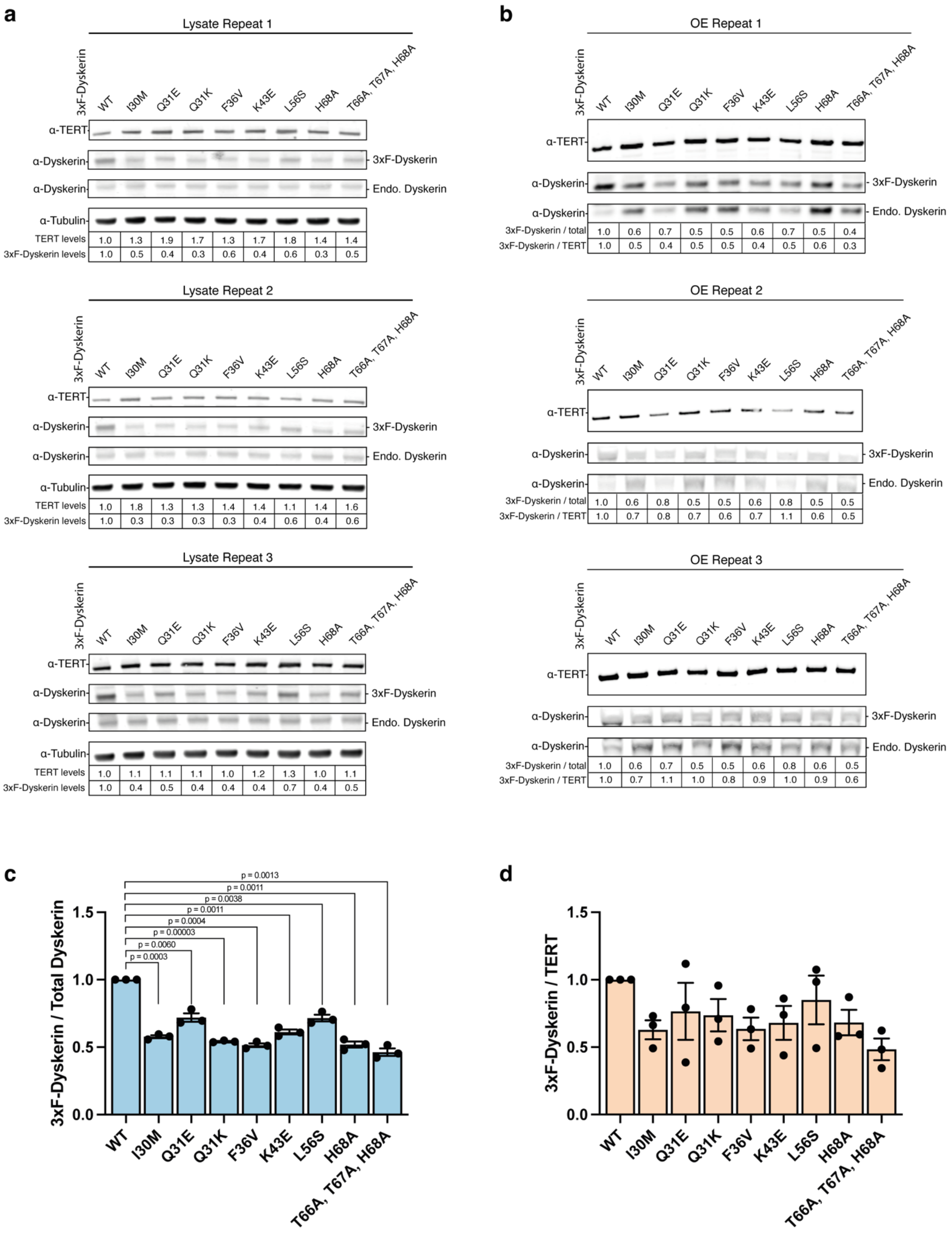

Supplementary Fig. 7: Biochemical characterisation of the wild-type (WT) and dyskerin telomerase

mutants. a Immunoblots of the crude lysates of cells overexpressed with TERT, hTR and 3xFLAG-tagged WT or mutant dyskerin, shown in triplicate. TERT and 3xFLAG-dyskerin levels are normalised to tubulin levels. The levels for dyskerin mutant samples are normalised to the WT sample. **b** Immunoblots of the elution from the oligonucleotide affinity purification (OE) for the WT and dyskerin mutants, shown in triplicate. Total dyskerin levels are calculated as a sum of 3xFLAG-dyskerin and endogenous (Endo.) dyskerin levels. The ratios of 3xFLAG-dyskerin over the total dyskerin levels and of 3xFLAG-dyskerin over TERT levels are normalised to the WT sample. **c** Bar graph of the ratios of 3xFLAG-dyskerin over the total amount of dyskerin for WT and dyskerin mutants, shown in (**b**). Significant p values are reported. **d** Bar graph of the ratios between the 3xFLAG-dyskerin and TERT, calculated in (**b**). The experiments in (**c, d**) were performed in triplicate and all three replicates are shown. Error bars represent the standard error of the mean (SEM) from n=3 independent replicates. Source data for this figure are provided as a Source Data file.

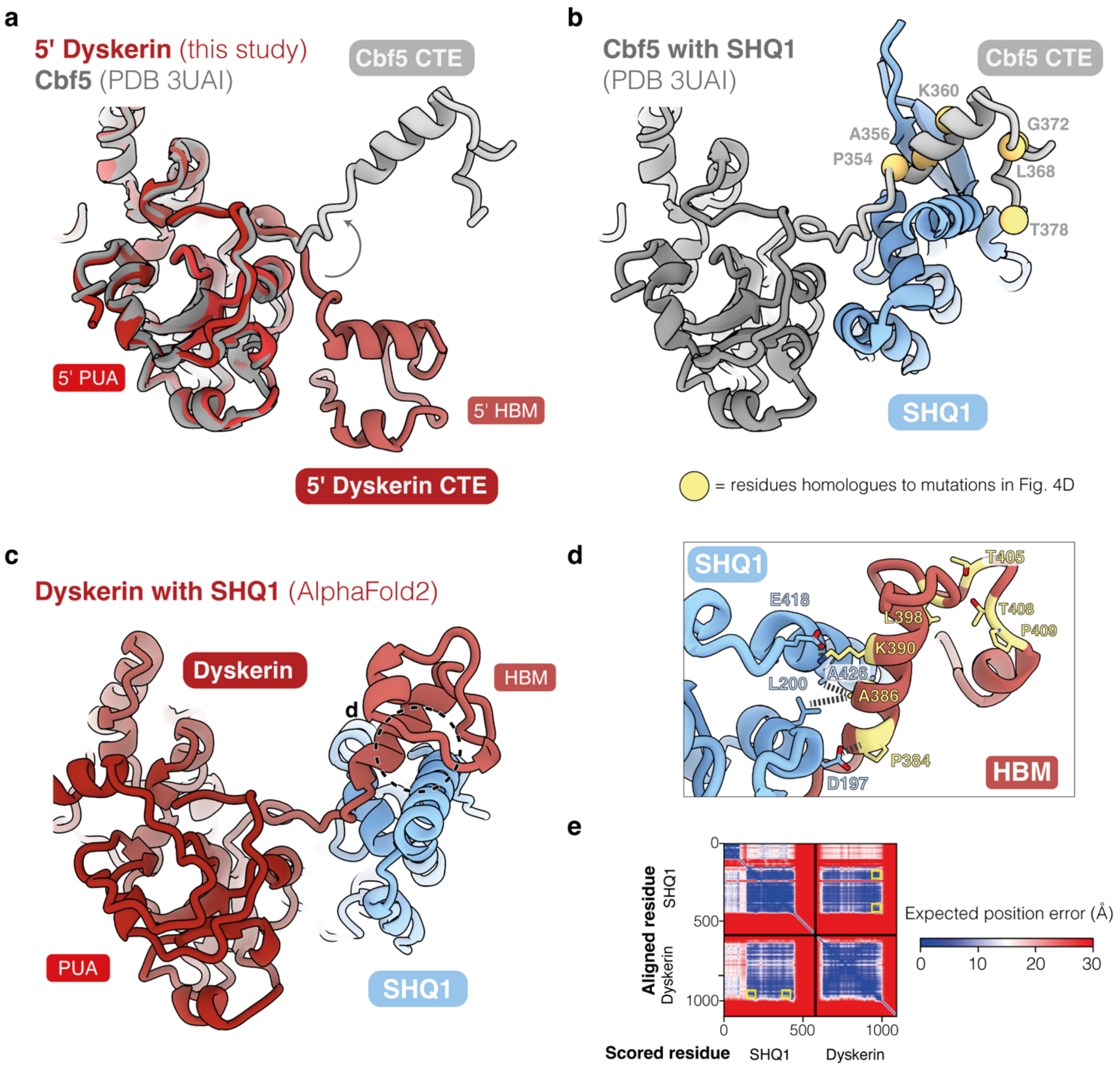

Supplementary Fig. 8: Cbf5 (dyskerin) interaction with the assembly factor SHQ1. a Comparison of the 5' dyskerin in our structure (red) and Cbf5 from *Saccharomyces cerevisiae* (*S. cerevisiae*) (grey) (PDB 3UAI, [https://doi.org/10.2210/pdb3UAI/pdb])[4]. The structures were aligned on the PUA domain. The most prominent difference is observed between the CTE of the 5' dyskerin (at the 5' HBM) (also see Fig. 4c) and the CTE of Cbf5. The arrow indicates the difference in position observed between the CTE of *S. cerevisiae* Cbf5 and the 5' dyskerin CTE. **b** Cbf5 from *S. cerevisiae* in complex with the assembly factor SHQ1 (blue) (PDB 3UAI, [https://doi.org/10.2210/pdb3UAI/pdb])[4]. The residues of Cbf5 that are equivalent to the disease mutations in human dyskerin (also see Fig. 4d) are shown as yellow spheres. These residues are involved in interaction with the SHQ1 in the *S. cerevisiae* structure. **c** AlphaFold2 prediction of human dyskerin with human SHQ1[5]. The HBM of dyskerin interacts with SHQ1 in a similar manner as observed

in the *S. cerevisiae* Cbf5-SHQ1 complex in (**b**). The location of interactions shown in (**d**) is indicated with a circle. **d** Close-up view of the interaction predicted in (**c**)[5]. Disease-associated residues of the HBM of dyskerin are shown as yellow sticks (also see Fig. 4d). SHQ1 residues and their predicted interactions with the dyskerin disease mutants are shown. **e** 2D plot of Predicted Aligned Error (PAE) depicting the AlphaFold2 model confidence in interaction between SHQ1 and dyskerin. The area of interaction described in (**d**) is marked with yellow squares. The expected error is expressed as expected position error in Å.

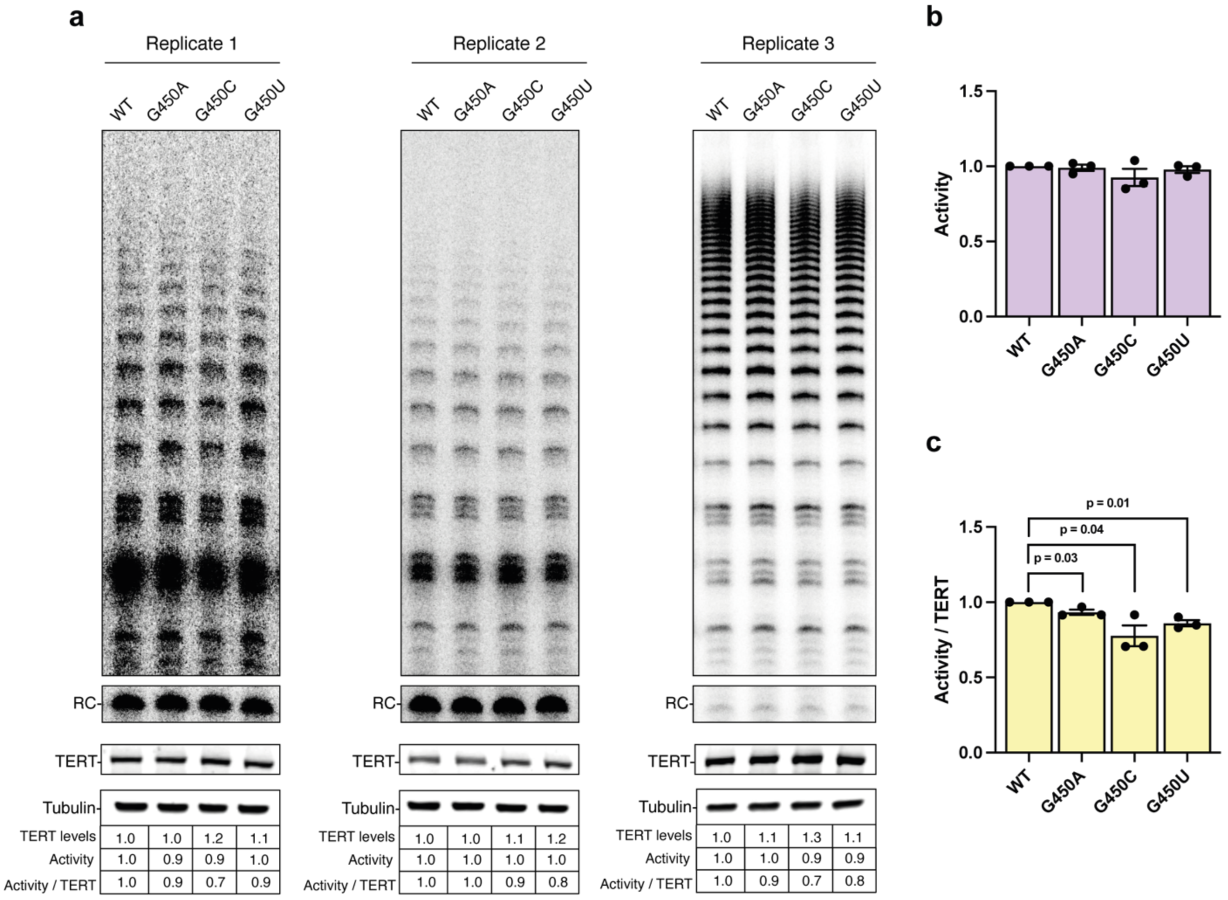

Supplementary Fig. 9: Biochemical characterisation of the wild-type (WT) and G450 hTR telomerase mutants. a Telomerase activity assays and immunoblots of the crude cell lysates of cells expressing WT and hTR mutants (G450A, G450C and G450U). The experiments were performed in triplicate and all three replicates were shown. TERT levels are normalised to tubulin levels, while telomerase activity is normalised to a radioactively labelled oligonucleotide recovery control (RC). A ratio of telomerase activity to TERT levels is calculated for each replicate. TERT levels, telomerase activity and activity per TERT are reported with the values normalised to the WT sample. **b** Bar graph of relative telomerase activity for WT and G450 mutant hTR samples shown in (**a**). **c** Bar graph of relative telomerase activity per TERT levels for WT and G450 mutant hTR samples shown in (**a**). Significant p values are reported. Error bars represent standard error of the mean (SEM) from n=3 independent replicates. Source data for this figure are provided as a Source Data file.

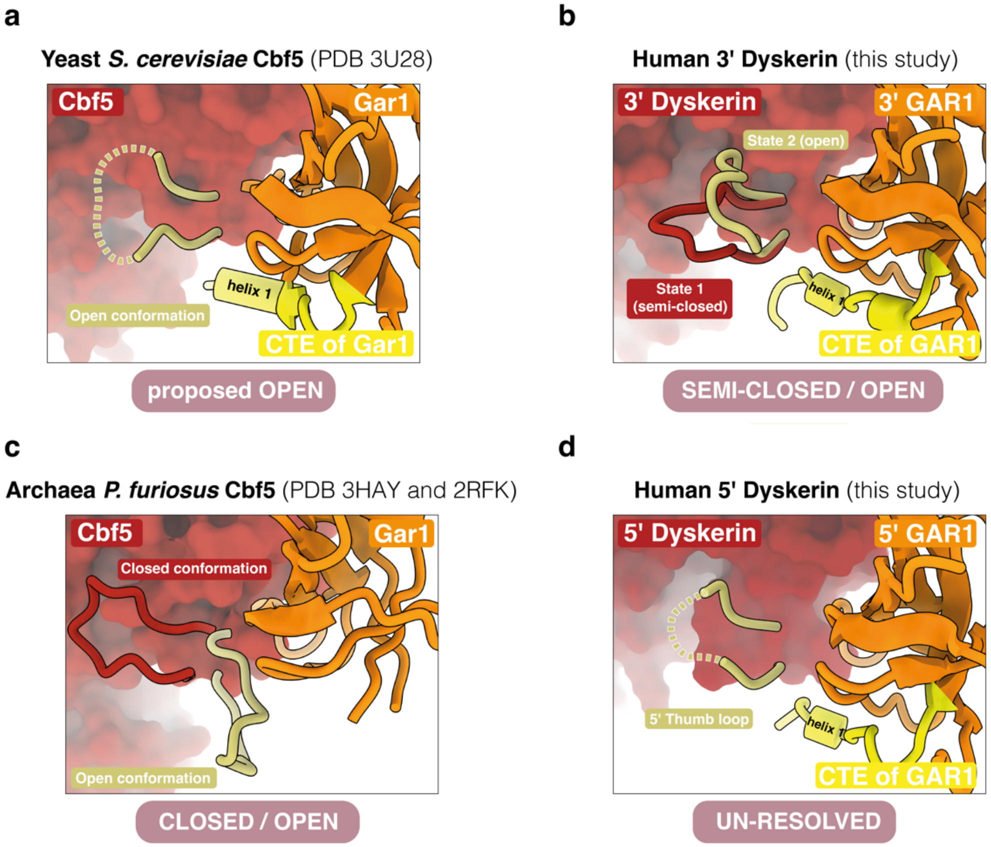

Supplementary Fig. 10: Comparison of the thumb loops from human dyskerin and Cbf5 (yeast and archaea). a The partially resolved yeast thumb loop in a proposed open conformation with Gar1 (PDB 3U28 [https://doi.org/10.2210/pdb3U28/pdb])[6]. Unresolved residues are depicted with a dashed olive line. The C-terminal helix 1 of yeast Gar1, analogous to helix 1 of human GAR1, is labelled. **b** An overlay of the two conformations we resolve in this paper: the semi-closed conformation (red) and the open conformation (olive) of the fully resolved 3' dyskerin thumb loop with the 3' GAR1. Helix 1 of the 3' GAR1 is labelled. **c** A fully resolved archaeal thumb loop in a closed (red) and an open (olive) conformation with Gar1 (PDB 3HAY [https://doi.org/10.2210/pdb3HAY/pdb] and 2RFK [https://doi.org/10.2210/pdb2rfk/pdb])[7,8]. Archaeal Gar1 lacks the C-terminal helix analogous to helix 1of eukaryotic GAR1. **d** The partially resolved thumb loop on the 5' dyskerin in our structure. Helix 1 of the 5' GAR1 that is positioned in a similar manner to the one in (**b**) is labelled.

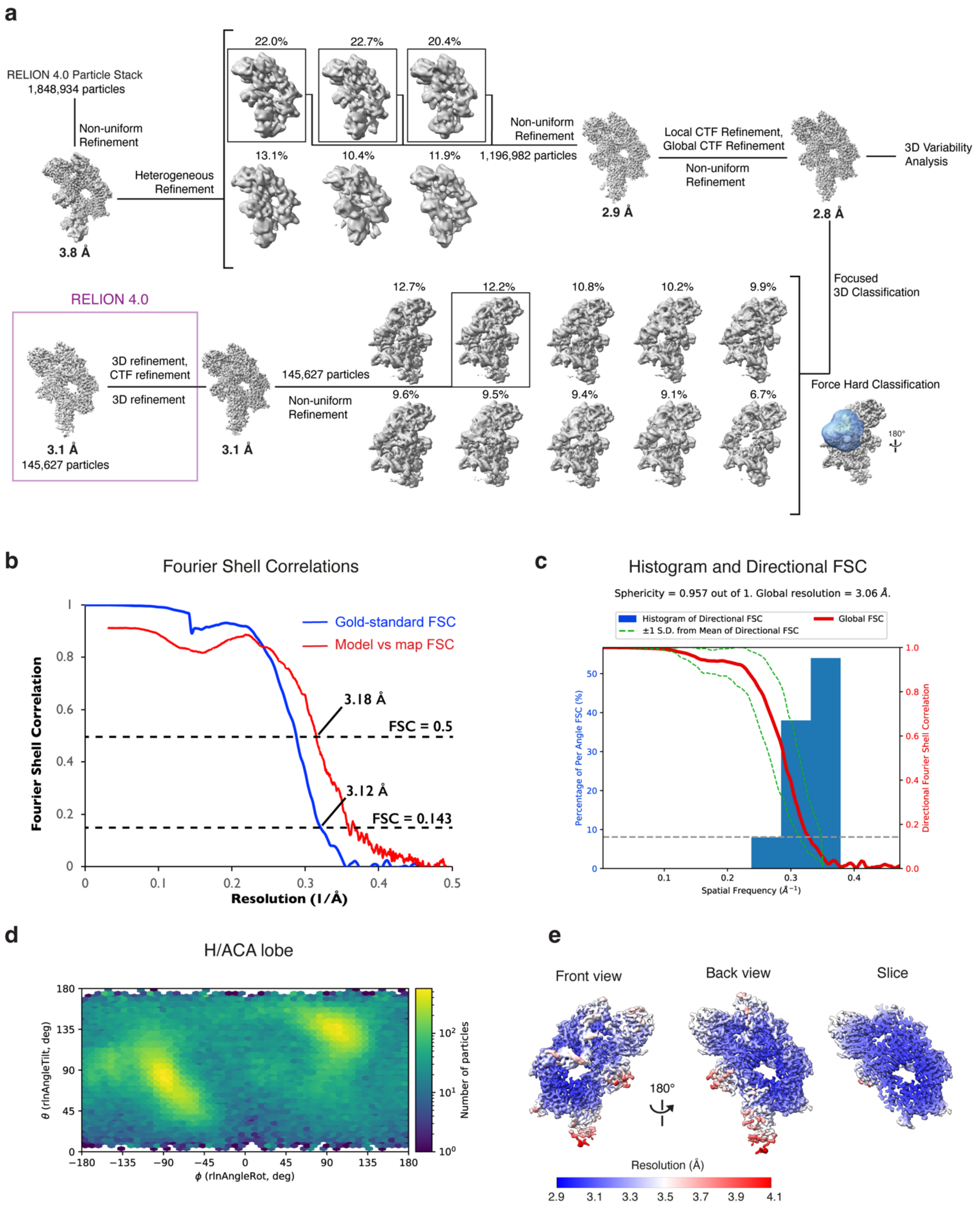

Supplementary Fig. 11: The 3.1 Å reconstruction of the telomerase H/ACA RNP lobe with the thumb loop of the 3' dyskerin in an open conformation. a Image processing workflow yielding the 3.1 Å reconstruction of telomerase H/ACA RNP. **b** Gold-standard (blue) and model-vs-map (red) FSC plots for

the telomerase H/ACA RNP reconstruction. Resolution was estimated at FSC = 0.143 (gold-standard) and at FSC = 0.5 (model-vs-map). **c** Directional FSC plots and sphericity values for the 3.1 Å telomerase H/ACA RNP reconstruction. The 3D-FSC server (https://3dfsc.salk.edu/) was used to calculate directional FSC plots. **d** 2D histograms displaying the Euler angles covered by particles from the reconstruction. A Python script (https://githubhelp.com/Guillawme/angdist) was used to calculate the 2D histograms. **e** Local resolution plot for the 3.1 Å telomerase H/ACA RNP obtained by RELION[1]. The position of the thumb loop of the 3' dyskerin is indicated with a circle.

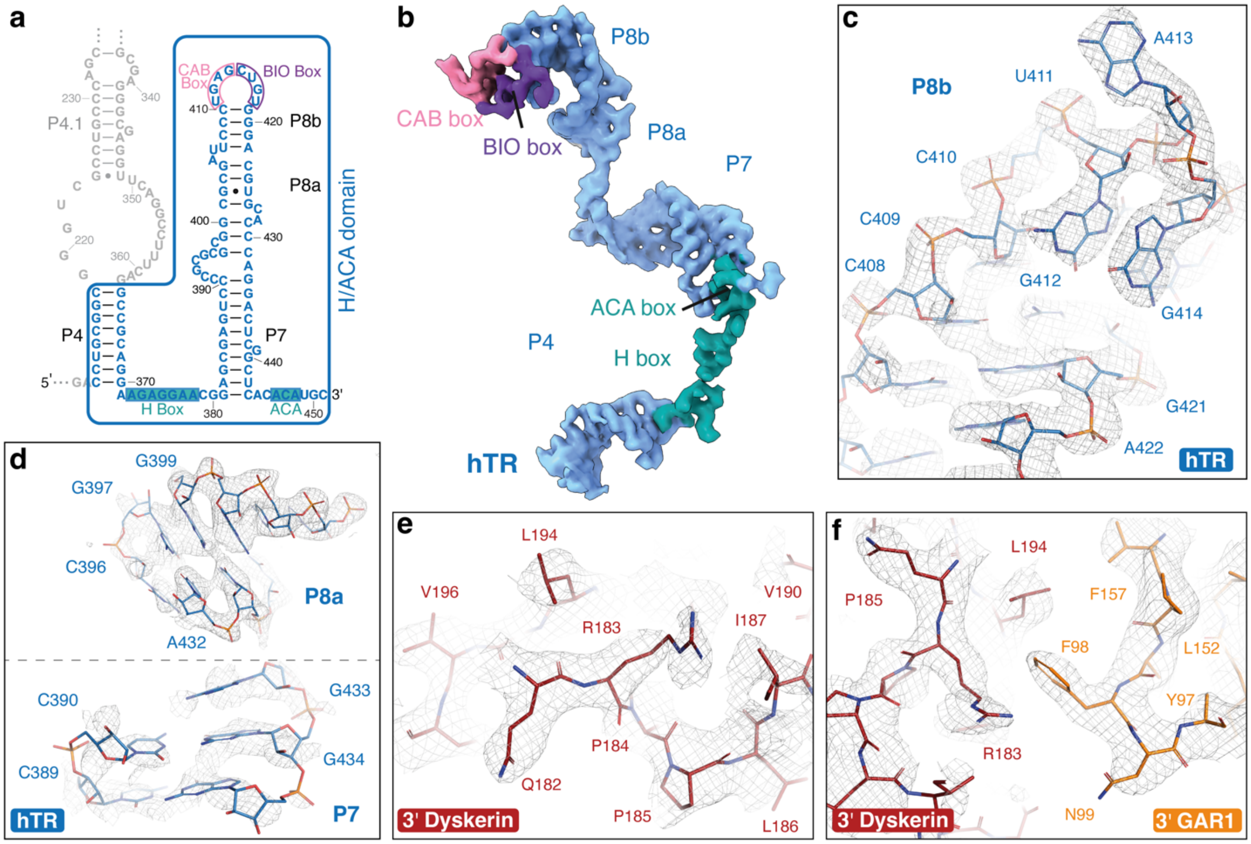

Supplementary Fig. 12: Representative cryo-EM densities of the 3.1 Å telomerase H/ACA RNP map. **a** Secondary structure of the H/ACA domain of hTR in the 3.1 Å telomerase H/ACA RNP map. **b** Full cryo-EM density of hTR. **c, d** Close-up view of the hTR fit into the cryo-EM density. P7, P8a and P8b stem-loops of hTR are shown. **e** The 3' dyskerin thumb loop (also see Fig. 8e). **f** Interactions between the 3' dyskerin thumb loop and the 3' GAR1 (also see Fig. 8d, e). Residue F98 of the 3' GAR1 that undergoes a rotameric switch is shown.

Supplementary Table 1: Cryo-EM data collection, refinement, and validation statistics.

	**Telomerase H/ACA RNP semi-closed state**<br>EMD-17190<br>PDB 8OUE	**Telomerase H/ACA RNP open state**<br>EMD-17191<br>PDB 8OUF
**Data collection and Processing**	**Dataset 1/Dataset 2**	
Microscope	Titan Krios G3i	
Voltage (keV)	300	
Camera	Gatan K3	
Magnification	81,000	
Pixel size at detector (Å/pixel)	1.059	
Total electron exposure ($e^{-}/Å^2$)	47-50	
Exposure rate (e-/ $Å^2$/sec)	15.66	
Number of frames	48	
Defocus range (µm)	1.0-3.0	
Automation software	EPU	
Energy filter slit width	20 eV	
Micrographs collected (no.)	4,912 / 36,141	
Total extracted particles (no.)	1,367,230/17,377,762	
**<u>For each reconstruction:</u>**		
Final particles (no.)	199,360	145,627
Point-group	C1	C1
Estimated error (rotations/translations)	1.691/0.74229	2.106/0.880
Resolution (global, Å)	2.72	3.12
FSC 0.5 (unmasked/masked)	3.06/2.90	3.32/3.18
FSC 0.143 (unmasked/masked)	2.56/2.45	2.85/2.78
Resolution range (local, Å)	2.7-5.3	3.0-7.5
3DFSC Sphericity	0.954	0.957
Map sharpening *B* factor ($Å^2$)	-61.36	-103.508
Map sharpening methods	RELION 4.0	RELION 4.0
**Model composition**		
Protein (residues)	1674	1674
RNA/DNA (nucleotides)	92	86
**Model Refinement**		
Refinement package	REFMAC5.8	PHENIX/REFMAC5.8
- real or reciprocal space	Reciprocal Space	Real/Reciprocal Space
- resolution cutoff	0.5	0.5
Model-Map scores		
- CCvolume/mask	0.83/0.84	0.86/0.87
B factors ($Å^2$)		
Protein residues (min/max/mean)	75.46/270.1/144.39	50.68/231.66/121.88
RNA/DNA (min/max/mean)	86.89/266.35/155.21	59.56/249.64/133.06
R.m.s. deviations from ideal values		
Bond lengths (Å) (#>4σ)	0.007 (0)	0.006 (0)
Bond angles (°) (#>4σ)	1.094 (0)	1.096 (1)
**Validation**		
MolProbity score	0.76	0.93
CaBLAM outliers (%)	1.05	1.42
Clashscore	0.84	1.75
Poor rotamers (%)	0.27	0.14
C-beta deviations (%)	0.00	0.00
Q-score (protein/nucleic)	0.681/0.651	0.652/0.603
EMRinger score	4.46	4.67
Ramachandran plot		
Favored (%)	98.12	99.09
Outliers (%)	0.00	0.00

Supplementary Table 2: Modelling of protein and RNA components.

	Subunit	Total residues	M.W. (kDa)	Modelled	Chain name
Telomerase H/ACA RNP semi-closed state	hTR	451	146	211-218, 362-393, 399-438, 440-450	B
	5'-dyskerin	514	57	23-186, 192-421	C
	5'-GAR1	217	21	66-159	D
	5'-NHP2	153	17	23-152	E
	5'-NOP10	64	8	1-64	F
	3'-dyskerin	514	57	43-395	G
	3'-GAR1	217	21	65-161	H
	3'-NHP2	153	17	23-152	I
	3'-NOP10	64	8	1-64	J
	TCAB1	548	59	146-204, 209-443, 449-489, 510-522	K
Telomerase H/ACA RNP open state	hTR	451	146	211-218, 362-390, 396-397, 399-404, 407-426, 429-438, 440-450	B
	5'-dyskerin	514	57	23-186, 192-421	C
	5'-GAR1	217	21	66-159	D
	5'-NHP2	153	17	23-152	E
	5'-NOP10	64	8	1-64	F
	3'-dyskerin	514	57	43-395	G
	3'-GAR1	217	21	65-161	H
	3'-NHP2	153	17	23-152	I
	3'-NOP10	64	8	1-64	J
	TCAB1	548	59	146-204, 209-443, 449-489, 510-522	K

SUPPLEMENTARY REFERENCES

1. Kimanius, D., Dong, L., Sharov, G., Nakane, T. & Scheres, S. H. W. New tools for automated cryo-EM single-particle analysis in RELION-4.0. *Biochem J* 478, 4169–4185 (2021).

2. Sievers, F. *et al.* Fast, scalable generation of high-quality protein multiple sequence alignments using Clustal Omega. *Mol Syst Biol* 7, 539–539 (2011).

3. Robert, X. & Gouet, P. Deciphering key features in protein structures with the new ENDscript server. *Nucleic Acids Res* 42, W320–W324 (2014).

4. Li, S., Duan, J., Li, D., Ma, S. & Ye, K. Structure of the Shq1–Cbf5–Nop10–Gar1 complex and implications for H/ACA RNP biogenesis and dyskeratosis congenita. *Embo J* 30, 5010–5020 (2011).

5. Jumper, J. *et al.* Highly accurate protein structure prediction with AlphaFold. *Nature* 596, 583–589 (2021).

6. Li, S. *et al.* Reconstitution and structural analysis of the yeast box H/ACA RNA-guided pseudouridine synthase. *Gene Dev* 25, 2409–2421 (2011).

7. Liang, B., Xue, S., Terns, R. M., Terns, M. P. & Li, H. Substrate RNA positioning in the archaeal H/ACA ribonucleoprotein complex. *Nat Struct Mol Biol* 14, 1189–1195 (2007).

8. Duan, J., Li, L., Lu, J., Wang, W. & Ye, K. Structural Mechanism of Substrate RNA Recruitment in H/ACA RNA-Guided Pseudouridine Synthase. *Mol Cell* 34, 427–439 (2009).
