# Supplementary material for: 2.7 Å cryo-EM structure of human telomerase H/ACA ribonucleoprotein
Source: Nat Commun. 2024 Jan 25;15:746. doi: 10.1038/s41467-024-45002-x (PMC10811338; doi:10.1038/s41467-024-45002-x)
Supplement: Supplementary file 7 — Reporting Summary [file 41467_2024_45002_MOESM7_ESM.pdf]

nature portfolio

Corresponding author(s): THI HOANG DUONG NGUYEN

Last updated by author(s): Dec 1, 2023

Reporting Summary

Nature Portfolio wishes to improve the reproducibility of the work that we publish. This form provides structure for consistency and transparency in reporting. For further information on Nature Portfolio policies, see our Editorial Policies and the Editorial Policy Checklist.

Statistics

For all statistical analyses, confirm that the following items are present in the figure legend, table legend, main text, or Methods section.

n/a	Confirmed	
☒	☐	The exact sample size (*n*) for each experimental group/condition, given as a discrete number and unit of measurement
☐	☒	A statement on whether measurements were taken from distinct samples or whether the same sample was measured repeatedly
☐	☒	The statistical test(s) used AND whether they are one- or two-sided<br>*Only common tests should be described solely by name; describe more complex techniques in the Methods section.*
☒	☐	A description of all covariates tested
☒	☐	A description of any assumptions or corrections, such as tests of normality and adjustment for multiple comparisons
☐	☒	A full description of the statistical parameters including central tendency (e.g. means) or other basic estimates (e.g. regression coefficient) AND variation (e.g. standard deviation) or associated estimates of uncertainty (e.g. confidence intervals)
☐	☒	For null hypothesis testing, the test statistic (e.g. F, t, r) with confidence intervals, effect sizes, degrees of freedom and P value noted<br>*Give P values as exact values whenever suitable.*
☒	☐	For Bayesian analysis, information on the choice of priors and Markov chain Monte Carlo settings
☒	☐	For hierarchical and complex designs, identification of the appropriate level for tests and full reporting of outcomes
☒	☐	Estimates of effect sizes (e.g. Cohen's *d*, Pearson's *r*), indicating how they were calculated

Our web collection on statistics for biologists contains articles on many of the points above.

Software and code

Policy information about availability of computer code

Data collection	EPU software for cryo-EM data collection
Data analysis	Cryo-EM data analysis: RELION 4.0, CryoSPARC v4.1.2. Structural analysis and building: Chimera, ChimeraX 1.4, Pymol 2.1, Coot 0.9.8.1, AlphaFold2, REFMAC5.8, ISOLDE, ERRASER ROSIE-1, PHENIX 1.20, PROSMART, LIBG, MolProbity; Gel images were analyzed using ImageJ, Microsoft Excel, Prism Graphpad, ImageQuant.

For manuscripts utilizing custom algorithms or software that are central to the research but not yet described in published literature, software must be made available to editors and reviewers. We strongly encourage code deposition in a community repository (e.g. GitHub). See the Nature Portfolio guidelines for submitting code & software for further information.

Data

Policy information about availability of data

All manuscripts must include a data availability statement. This statement should provide the following information, where applicable:

- Accession codes, unique identifiers, or web links for publicly available datasets
- A description of any restrictions on data availability
- For clinical datasets or third party data, please ensure that the statement adheres to our policy

The cryo-EM maps of the telomerase H/ACA RNP semi-closed state and open state have been deposited in the Electron Microscopy Database (EMDB) under accession codes EMD-17190 and EMD-17191, respectively. PDB coordinates for the telomerase H/ACA RNP lobe semi-closed state and open state have been

deposited in the Protein Data Bank under accession codes 8OUE [https://doi.org/10.2210/pdb8OUE/pdb] and 8OUF [https://doi.org/10.2210/pdb8OUF/pdb], respectively. Pymol sessions of the deposited PDB models are also included in Supplementary Data 1 and 2 in Additional Supplementary files. The source data underlying Supplementary Figs. 7a, b. 9a are provided as a Source Data file. Source data are provided with this paper. Materials are available from T.H.D.N. under a material transfer agreement with the MRC Laboratory of Molecular Biology. Correspondence should be addressed to T.H.D.N. (knguyen@mrc-lmb.cam.ac.uk).

Research involving human participants, their data, or biological material

Policy information about studies with human participants or human data. See also policy information about sex, gender (identity/presentation), and sexual orientation and race, ethnicity and racism.

Reporting on sex and gender	NA
Reporting on race, ethnicity, or other socially relevant groupings	NA
Population characteristics	NA
Recruitment	NA
Ethics oversight	NA

Note that full information on the approval of the study protocol must also be provided in the manuscript.

Field-specific reporting

Please select the one below that is the best fit for your research. If you are not sure, read the appropriate sections before making your selection.

☒ Life sciences ☐ Behavioural & social sciences ☐ Ecological, evolutionary & environmental sciences

For a reference copy of the document with all sections, see nature.com/documents/nr-reporting-summary-flat.pdf

Life sciences study design

All studies must disclose on these points even when the disclosure is negative.

Sample size	Cryo-EM data were collected over approximately 7 days, yielding the number of particles required for extensive classification as described to achieve interpretable maps presented here. We determined the size of the dataset by extrapolating the number of particles required to achieve the target resolution using a small dataset.
Data exclusions	Through 2D and 3D classification procedures, we discarded "bad particles" or classes of particles that did not result in good 3D reconstruction. This procedure is standard in the EM field.
Replication	Our biochemical purification and activity assays are all performed in triplicate successfully. We include the triplicates in the Supplemental Information and the Source Data.
Randomization	Cryo-EM data were collected automatically and we process all the data collected. Therefore, randomization was not relevant to cryo-EM work presented here.
Blinding	Blinding was not relevant for the structure determination by cryo-EM. Cryo-EM data were collected automatically and we process all the data collected.

Reporting for specific materials, systems and methods

We require information from authors about some types of materials, experimental systems and methods used in many studies. Here, indicate whether each material, system or method listed is relevant to your study. If you are not sure if a list item applies to your research, read the appropriate section before selecting a response.

Materials & experimental systems

n/a	Involved in the study
☐	☒ Antibodies
☐	☒ Eukaryotic cell lines
☒	☐ Palaeontology and archaeology
☒	☐ Animals and other organisms
☒	☐ Clinical data
☒	☐ Dual use research of concern
☒	☐ Plants

Methods

n/a	Involved in the study
☒	☐ ChIP-seq
☒	☐ Flow cytometry
☒	☐ MRI-based neuroimaging

Antibodies

Antibodies used	rabbit anti-dyskerin antibody, Santa Cruz Biotechnology, Cat# sc-48794, lot E0214, RRID: AB_2091314<br>mouse anti-alpha tubulin, ProteinTech, Cat# 66031-1-Ig, lot 10004185<br>goat anti-rabbit Alexa-Fluor 680, Abcam Cat# ab175773, lot GR222353-8<br>goat anti-mouse Alexa-Fluor 680, Abcam Cat# ab175775, lot GR3273649-2
Validation	rabbit anti-dyskerin antibody, Western blots against various human lysates<br>mouse anti-alpha tubulin, Western blots against various human lysates<br>goat α-rabbit Alexa-Fluor 680 (Abcam)-Western blot against IgG<br>goat α-mouse Alexa-Fluor 680 (Abcam)-Western blot against IgG

Eukaryotic cell lines

Policy information about cell lines and Sex and Gender in Research

Cell line source(s)	HEK293T cells (ATCC, Cat# CRL-3216, RRID:CVCL_0063), Expi293FTM (ThermoFisher, Cat# A14527) and Spodoptera frugiperda (Sf9, Oxford Expression Technologies Ltd, Cat# 600100) were used for protein production
Authentication	None of the cell lines used were authenticated. This was obtained from a commercial source.
Mycoplasma contamination	Cells were not tested for mycoplasma contamination.
Commonly misidentified lines<br>(See ICLAC register)	No commonly misidentified lines were used

Plants

Seed stocks	NA
Novel plant genotypes	NA
Authentication	NA
